# Different Leaf Strategies Between Lithophytic and Terrestrial Orchids in a Subtropical Karst Forest

**DOI:** 10.3390/plants14081161

**Published:** 2025-04-08

**Authors:** Mei Yang, Dan Sun, Xiaoyin Wang, Shidan Zhu, Uromi Manage Goodale

**Affiliations:** 1 Guangxi Key Laboratory of Forestry Ecology and Conservation, College of Forestry, Guangxi University, Nanning 530004, China; yangmei108@outlook.com (M.Y.); wang.xiaoyin@outlook.com (X.W.); 2 Nanning Institute of Tropical Botany, Nanning Botanical Garden, Nanning 530029, China; 3 Guangxi Key Laboratory of Beibu Gulf Marine Biodiversity Conservation, College of Marine Sciences, Beibu Gulf University, Qinzhou 535011, China; sundan0991@163.com; 4 Department of Health and Environmental Science, School of Science, Xian Jiaotong Liverpool University, Suzhou 215123, China; 5 Seed Conservation Specialist Group, Species Survival Commission, International Union for Conservation of Nature, 1196 Gland, Switzerland

**Keywords:** biomechanics, leaf saturated water content, nitrogen allocation, Orchidaceae

## Abstract

The leaf economic spectrum framework explains how plants optimize leaf traits for productivity, distribution, and stress tolerance. Orchids in Southwestern China’s karst forests, especially lithophytic species, are challenged by prolonged drought and limited light availability. This study investigated different leaf strategies between lithophytic and terrestrial orchids under the harsh karst environment. We measured key leaf traits, including photosynthesis, structure, biomechanics, nitrogen allocation, and water relations, in twenty-two lithophytic and six terrestrial orchids in a subtropical karst forest. After accounting for phylogenetic influences, we found that lithophytic orchids had a higher leaf mass per area, cuticle thickness, and biomechanical resistance (*F*_p_) but a lower maximum photosynthetic rate (A_max-mass_), nitrogen allocation to photosynthesis (N_T_), and saturated water content (SWC) than terrestrial orchids. These results suggest that lithophytic orchids prioritize structural investment and stress tolerance over photosynthetic efficiency. Across species, N_T_ correlated positively with A_max-mass_ and negatively with *F*_p_, highlighting nitrogen allocation as a key mechanism in leaf cost–benefit strategies. Additionally, SWC emerged as a critical driver of variation in multiple traits, supporting its integration into the leaf economic spectrum for orchids in karst ecosystems. This study offers new insights into orchid adaptation in subtropical karst environments, with implications for plant resilience under changing climates.

## 1. Introduction

Leaves are essential for photosynthesis and form the foundation of terrestrial food webs [1]. Their traits vary widely due to evolutionary adaptations [2], which are systematically analyzed using the leaf economic spectrum (LES). It links key leaf traits along a continuum of ecological strategies, balancing resource acquisition and conservation [1,3]. This framework has been widely applied to explore plant productivity, distribution, and stress tolerance in diverse ecosystems. Several previous studies have proposed that nitrogen allocation drives the trade-off between photosynthetic capacity and stress tolerance [4,5], implying a physiological basis underlying the LES [6]. However, sufficient experimental evidence was lacking. Water relation traits are crucial for studying plants’ drought resilience and could be incorporated into the LES [7]. An improved LES would enhance our understanding of plant environmental adaptation [8].

The karst landform (mainly limestone) in Southwestern China is characterized by shallow soils with low water holding capacity [9,10], high calcium and magnesium levels [11], and long dry seasons [12]. The karst ecosystem hosts diverse and endangered flora [13], particularly orchids [14]. The orchids usually grow in the understory of the karst forest, facing dual stresses of low light and drought. Several studies have indicated that orchids with leaf traits such as high leaf mass per unit area (LMA), thick leaves, and low epidermal conductance tend to adopt conservative leaf strategies that favour stress tolerance [15,16,17,18]. However, orchids are significantly underrepresented in the LES dataset, with limited data on critical traits such as nitrogen content, photosynthetic rates, and water relations [17]. Moreover, most orchids in karst forests are classified as lithophytes that grow on rock surfaces or crevices, but their adaptive strategies remain poorly understood. In comparison to terrestrial orchids (which grow in soil), the lithophytic orchids may have evolved distinct ecological strategies that are particularly well adapted for survival and reproduction in the karst rock environments. Furthermore, climate change may increase environmental pressure on karst ecosystems [19,20], threatening habitat-dependent orchids with limited dispersal and raising their extinction risk, particularly on isolated, dry limestone mountaintops [12,21]. Comparative studies of leaf traits between lithophytic and terrestrial orchids in karst ecosystems are critical for predicting how these species respond to environmental changes and for informing conservation strategies.

Hence, in this study, we selected 28 wild orchid species commonly found in a subtropical karst forest in the Northwest Guangxi Zhuang Autonomous Region, Southwestern China, and measured photosynthetic characteristics, leaf structure, biomechanics, nitrogen allocation, and water relations. The primary objective of this work was to explore the different leaf strategies between lithophytic and terrestrial orchids. Specifically, we hypothesized that lithophytic orchids, compared to their terrestrial counterparts, would adopt a more conservative ecological strategy with low photosynthetic capacity and high stress tolerance. This study would advance the understanding of orchid adaptations in karst ecosystems.

## 2. Materials and Methods

### 2.1. Study Site and Plant Materials

**Figure 1:**
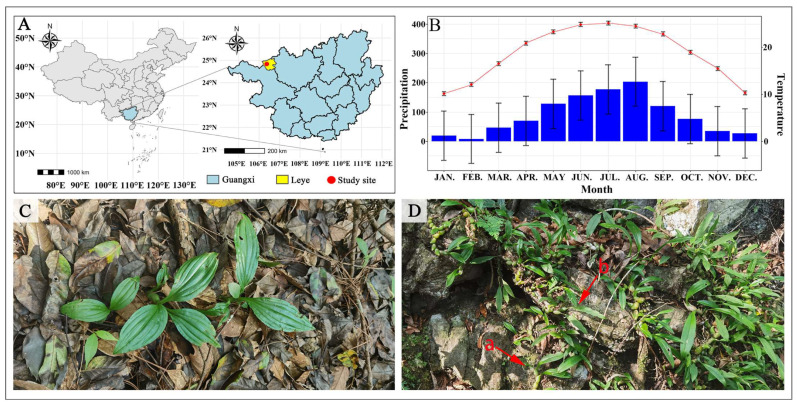
Location, average monthly temperature and precipitation, and the growth conditions of orchids in the study site. (**A**): Location of the study site. (**B**): Average monthly temperature and precipitation at the study site from 2013 to 2019. The red line is the temperature line, and the blue bars represent precipitation (Data source: Huaping Meteorological Station, Leye County Meteorological Bureau, Guangxi, China). Temperature and rainfall are measured in degrees Celsius and millimetres, respectively. (**C**): Terrestrial orchid: *Crepidium purpureum* in soil. (**D**): Lithophytic orchids: *Liparis esquirolii* (a) and *Panisea cavaleriei* (b) on the rocks.

The field study was conducted in the Guangxi Yachang National Nature Reserve (hereinafter referred to as Yachang Reserve), located between 24°44′16″ N and 24°53′58″ N latitude, and 106°11′31″ E–106°27′04″ E longitude [12] in Huaping Town, Leye County, Baise City, Northwestern Guangxi, Southwestern China (Figure 1A). The annual mean temperature in the study site was 18.8 °C, and the lowest and highest temperatures were −3 °C and 40.4 °C, respectively. The annual precipitation was 1065.4 mm (meteorological data sources: Leye County Meteorological Bureau, Huaping Town meteorological station), with rainfall concentrated between May and October, creating distinct wet and dry seasons. Field experiments were conducted in a karst limestone hilltop forest area at an altitude of approximately 950 m a.s.l. (Figure 1A). The vegetation at the study site is a mixed evergreen–deciduous broad-leaved forest with a canopy density exceeding 80%. Over 40 wild orchids inhabit the site, including 29 lithophytic, 9 terrestrial, 2 saprophytic, and 1 epiphytic. Terrestrial orchids grow in gentle depressions with shallow soils (Figure 1C), while lithophytic orchids are found attached to rock surfaces or cracks (Figure 1D). The soil properties of the growth media differ between lithophytic and terrestrial orchids. The lithophytic medium has a pH of 7.15, alkali-hydrolysable nitrogen content of 902.7 mg kg^−1^, and available phosphorus of 30.0 mg kg^−1^, whereas the terrestrial medium has a pH of 6.52, alkali-hydrolysable nitrogen content of 351.9 mg kg^−1^ and available phosphorus of 11.9 mg kg^−1^ [15]. Relative humidity ranged from > 80% in the morning to approximately 50% at 14:00.

In this study, 6 terrestrial and 22 lithophytic species were analyzed (Supplementary Table S1). The selection of more lithophytic orchids reflects their natural dominance (approximately 70% of local orchid species) in our study site. Moreover, since approximately 90% of local orchids are C_3_ plants [only three crassulacean acid metabolism (CAM) species] [15,22], this study excluded species that exhibit CAM pathway. 

### 2.2. Photosynthetic Parameters

Photosynthetic parameters were measured using a portable photosynthesis system with a red–blue light source chamber (LI COR-6400XT, LI-COR, Lincoln, NE, USA). Measurements were conducted on healthy, fully mature leaves of orchids in their vegetative growth phase between 09:00 and 14:00 on sunny days from June to September 2022. No environmental stress was induced during measurements.

Net photosynthetic rate (A_n_) was measured at 1200, 1000, 800, 600, 500, 400, 300, 200, 150, 100, 80, 50, 20, and 0 μmol photons m^−2^ s^−1^ photosynthetic photon flux density (PPFD), with the reference CO_2_ concentration set at 400 μmol mol^−1^. The A_n_-PPFD curve was fitted using a non-rectangular hyperbolic model [23] to determine the saturation light intensity (saturated PPFD) of each species. The photosynthetic response to intercellular CO_2_ concentrations (C_i_) was measured at the saturated PPFD for each species, with CO_2_ concentrations set at 400, 300, 200, 100, 50, 400, 600, 800, 1000, 1200, 1500, and 1800 μmol mol^−1^. The A_n_-C_i_ curve was fitted according to Farquhar et al. [24] to obtain the area-standardized maximum carboxylation (V_cmax_) and electron transport rates (J_max_).

The maximum photosynthetic rate per unit leaf area (A_max-area_) and stomatal conductance (g_s_) was measured under saturated PPFD and a reference CO_2_ concentration of 400 μmol mol^−1^. Prior to measurement, leaves were exposed to saturated PPFD until stomatal conductance reached a stable maximum. Photosynthetic water-use efficiency (WUE) was calculated as WUE = A_max-area_/g_s_.

### 2.3. Leaf Structural, Water-Related Traits, and Nitrogen Content

Leaf samples were collected after measuring A_max-area_ for subsequent analysis of leaf traits. After collection, the samples were transported in iceboxes to the laboratory at Nanning Botanical Garden. Leaf area (LA) was measured using a scanner (e-STUDIO2618A, TOSHIBA, Tokyo, Japan) and analyzed with Image J 1.53e software (National Institutes of Health, Bethesda, MD, USA). Fresh weight was measured using an analytical balance (XJ220ASCS, Precisa, Zurich, Switzerland). After soaking in ultrapure water for 24 h, leaf saturated weight (SW) was recorded. The leaves were then dried at 65 ℃ for 48 h to constant weight to obtain the leaf dry mass (DM). The leaf mass per area (LMA) was calculated using the following formula: LMA = DM/LA. Leaf dry mass content (LDMC) was calculated as LDMC = DM/SW. Leaf-saturated water content (SWC) was calculated as SWC = (SW − DM)/DM. The mass standardized maximum photosynthetic rate (A_max-mass_) was calculated as A_max-mass_ = A_max-area_/LMA. The leaf nitrogen content (N_m_) was determined after grinding dried leaf samples from the same plant into a powder using an automatic Kjeldahl nitrogen analyser (KJELTEC8400, Foss, Copenhagen, Denmark). Photosynthetic nitrogen-use efficiency (PNUE) was calculated as PNUE = A_max-mass_/N_m_.

### 2.4. Leaf Anatomical Traits

Leaf anatomical traits were measured following the protocols used by Yang et al. [25]. Cross-sections of leaves were prepared manually, avoiding the middle vein and examined under a binocular light microscope (Leica DM2500, Munich, Germany). Five visual fields were captured per leaf. Image J 1.53e software (National Institutes of Health) was used to measure leaf thickness (LT) and upper cuticle thickness (CT). Leaf density (LD) was calculated as LD = LMA/LT. Stomatal density (SD) was determined by applying colourless nail polish to the lower epidermis of the middle of the leaves, which was peeled off after drying, and examined under a binocular light microscope (Leica DM2500).

### 2.5. Leaf Punch Force

The leaf punch force (*F*_P_), the maximum force required to penetrate the leaf, was measured using a small tension tester (ZQ 990, Sanker Precision Instrument Co. Ltd., Shanghai, China). The maximum force required for a 1 mm flat-ended cylindrical puncture needle to penetrate the leaf (avoiding the main and secondary veins) was recorded. The *F*_P_ was calculated using *F*_P_ = maximum penetration force/circumference of the needle [26].

### 2.6. Calculation of Leaf Nitrogen Allocated to Photosynthetic Apparatus

The nitrogen content of the photosynthetic apparatus was estimated using the empirical formulas [Equations (1)–(3) presented below] developed by Niinemets and Tenhunen [27]. The leaf nitrogen was partitioned into three components: the carboxylation system (mostly in Rubisco), bioenergetic components, and the light-harvesting system. The fraction of nitrogen allocated to each component was calculated based on

(1) $$P_{C}=\frac{V_{cmax}}{6.25\times V_{cr}\times LMA\times N_{m}}\;,$$

(2) $$P_{B}=\frac{J_{max}}{8.06\times J_{mc}\times LMA\times N_{m}},$$

(3) $$P_{L}=\frac{C_{c}}{C_{B}\times N_{m}},$$

where *P_C_*, *P_B_*, and *P_L_* are the fraction of leaf nitrogen allocated to the carboxylation system, bioenergetics components, and the light-harvesting system. *C_C_* (mmol g^−1^) is the leaf chlorophyll content (the experimental method for determination of chlorophyll content followed the protocols used by Li et al. [28]), *V_cr_* (μmol CO_2_ [g Rubisco]^−1^s^−1^] is the specific activity of Rubisco, *J_mc_* (μmol electrons [μmol cyt f]^−1^ s^−1^) is the activity of electron transport, and *C_B_* (mmol Chl [g *N_m_*]^−1^) is the ratio of chlorophyll to nitrogen in the light-harvesting system. *V_cr_*, *J_mc_*, and *C_B_* are calculated using the following formulae:

(4) $$V_{cr}=\frac{e^{\left(c-\frac{{∆H}_{a}}{R\times T_{k}}\right)}}{1+e^{\frac{∆S\times T_{k}-{∆H}_{d}}{R\times T_{k}}}},$$

(5) $$J_{mc}=\frac{e^{\left(c-\frac{{∆H}_{a}}{R\times T_{k}}\right)}}{1+e^{\frac{∆S\times T_{k}-{∆H}_{d}}{R\times T_{k}}}},$$

(6) $$C_{B}=1.94+\frac{12.6}{LMA},$$

where *c* is the proportional constant, *c*(*V_cr_*) = 32.9, *c*(*J_mc_*) *=* 14.77; Δ*H_a_* is the activation energy, Δ*H_a_*(*V_cr_*) *=* 74,000 J mol^−1^, Δ*H_a_*(*J_mc_*) = 24,100 J mol^−1^; *R* is the universal gas constant, *R* = 8.314(J K^−1^ mol^−1^); T_k_ (K) is the leaf Kelvin temperature, *T_k_* = 273.15 + t (centigrade temperature); *ΔS* is the entropy term, ∆S(V_cr_) = 645 J K^−1^ mol^−1^, ∆S(J_mc_) = 1810 J K^−1^ mol^−1^; and ΔH_d_ is the deactivation energy, ΔH_d_ (V_cr_) = 203,000 J mol^−1^, ΔH_d_(J_mc_) = 56,4150 J mol^−1^ [27]. The total fraction of leaf nitrogen allocated to the photosynthetic system (P_T_) was calculated using the following formula: P_T_ = P_C_ + P_B_ + P_L_; the total N_T_ content in the photosynthetic system was calculated using the following formula: N_T_ = N_m_ × P_T_.

### 2.7. Statistical Analyses

The results are presented as mean values of seven individuals of each sampled orchid species, with three or four leaves per individual. All analyses and graphics were conducted using R 4.3.2 (R Core Team, 2023 [RStudio, Boston, MA, USA]). Independence (Chi-square test), normal distribution (Shapiro test), and homogeneity tests (Bartlett test) of variance were performed for each variable before data analysis. As the data of all variables did not pass the normal distribution test and some data did not pass the homogeneity test of variance, the nonparametric Mann–Whitney test was used to analyze the differences between terrestrial and lithophytic orchid species. Data were Log-transformed before correlation analysis. A linear regression model was used to fit the correlation lines between relevant variables. The *F*-test was used to analyze the results of the linear regression model fitting line. Closely related species tend to have similar traits and residuals from the regression line, which may cause type I or II errors. To address this, we used phylogenetic generalized least squares (PGLS) analysis (“phytools” package) to account for phylogeny in trait correlation analysis. Analysis of covariance (ANCOVA) was used to test the homogeneity of regression and to determine whether the correlation between traits was consistent across different growth forms (“car” package). Principle component analysis (PCA) was used to detect trait correlations and their contributions to the overall variation in traits (“FactoMineR” and “factoextra” packages). Permutational multivariate analysis of variance (PERMANOVA) was used to detect significant differences in trait combinations between growth forms (“vegan” package). Prior to PERMANOVA analysis, collinear variables were removed through correlation matrix screening, and the dataset was standardized. Subsequently, a similarity percentage analysis (SIMPER) was conducted to determine the key variables contributing most significantly to the observed dissimilarities (“corrplot” and “caret” packages).

## 3. Results

### 3.1. Differences in Leaf Functional Traits Between Lithophytic and Terrestrial Orchids

Of the 16 traits examined, 12 exhibited significant differences between lithophytic and terrestrial orchids (Table 1).

**Figure 2:**
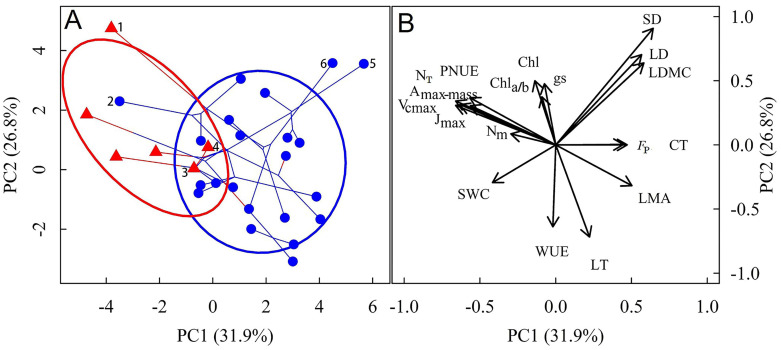
Principal component analysis (PCA) of the traits evaluated in the present study after excluding phylogenetic effects. (**A**): Loadings of terrestrial (red triangles) and lithophytic (blue dots) species along PCA axes; (**B**): The correlations between traits and principal components. A_max-mass_: mass-standardized maximum photosynthetic rate; V_cmax_: maximum carboxylation rate; J_max_: maximum electron transport rate; LMA: leaf mass per area; LDMC: leaf dry mass content; N_m_: leaf nitrogen content; N_T_: total nitrogen content in photosynthetic apparatus; PNUE: photosynthetic nitrogen-use efficiency; Chl: leaf chlorophyll content; Chl_a/b_: the ratio of Chl_a_ to Chl_b_; CT: upper cuticle thickness; LT: leaf thickness; SD: stomatal density; g_s_: stomatal conductance; WUE: water-use efficiency; SWC: leaf saturated water content; LD: leaf density; *F*_P_: leaf punch force. 1: *Bletilla striata*; 2: *Dendrobium chrysanthum*; 3: *Calanthe argenteostriata*; 4: *Calanthe triplicata*; 5: *Cymbidium floribundum*; 6: *Pholidota yunnanensis*.

We considered the effects of inter-species relatedness in PCA (Figure 2), and the results revealed that lithophytic and terrestrial orchids were distinctly separated into two groups (PERMANOVA: r^2^ = 0.20, *p* = 0.009). The first two PCA axes explained 58.7% of the variance. The first axis exhibited a significant negative correlation with N_T_, N_m_, and SWC, whereas the second axis was positively correlated with Chl but negatively correlated with LT. According to the SIMPER analysis, these traits showed statistically significant contributions to the differentiation between terrestrial and lithophytic orchids, collectively accounting for 55.3% of the total dissimilarity. Terrestrial orchids congregated in the negative and positive regions of the first and second axes, respectively, and had a higher photosynthetic capacity than that of lithophytic orchids. Furthermore, lithophytic orchids were mainly distributed in the region to the right of the terrestrial orchids and had higher values of LMA, LT, and *F*_P_ than those of the terrestrial orchids. Considering that multicollinearity among variables might lead to variance inflation and other statistical issues, we performed an additional PCA using only the retained variables after eliminating those with high collinearity (Figure S1). While this resulted in a moderate decrease in explained variance for the first two principal components (from 58.7% to 52.2%), the overall patterns remained consistent with our original findings.

Among the 22 lithophytic orchids, *Dendrobium chrysanthum* clustered with terrestrial orchids, exhibiting high A_max-mass_ and N_T_ and low LMA and *F*_P_. While *Cymbidium floribundum* and *Pholidota yunnanensis* were distinctly separate from the other 20 lithophytic species, characterized by extremely high LD, LDMC, SD, and low SWC. Among terrestrial orchids, *Bletilla striata* was distant from other terrestrial orchids, showing relatively high photosynthetic capacity and low LMA. Two terrestrial orchids, *Calanthe argenteostriata* and *Calanthe triplicata*, clustered close to the lithophytic group, with traits such as A_max-mass_, N_T_, SWC, LMA, LT, and *F*_P_, intermediate between those of lithophytic and other terrestrial orchids. These two species had the largest and lowest N_m_ and PNUE, respectively (Figure 2).

### 3.2. Correlations Between Leaf Nitrogen Allocation, Photosynthetic Characteristics, and Biomechanical Traits

**Figure 3:**
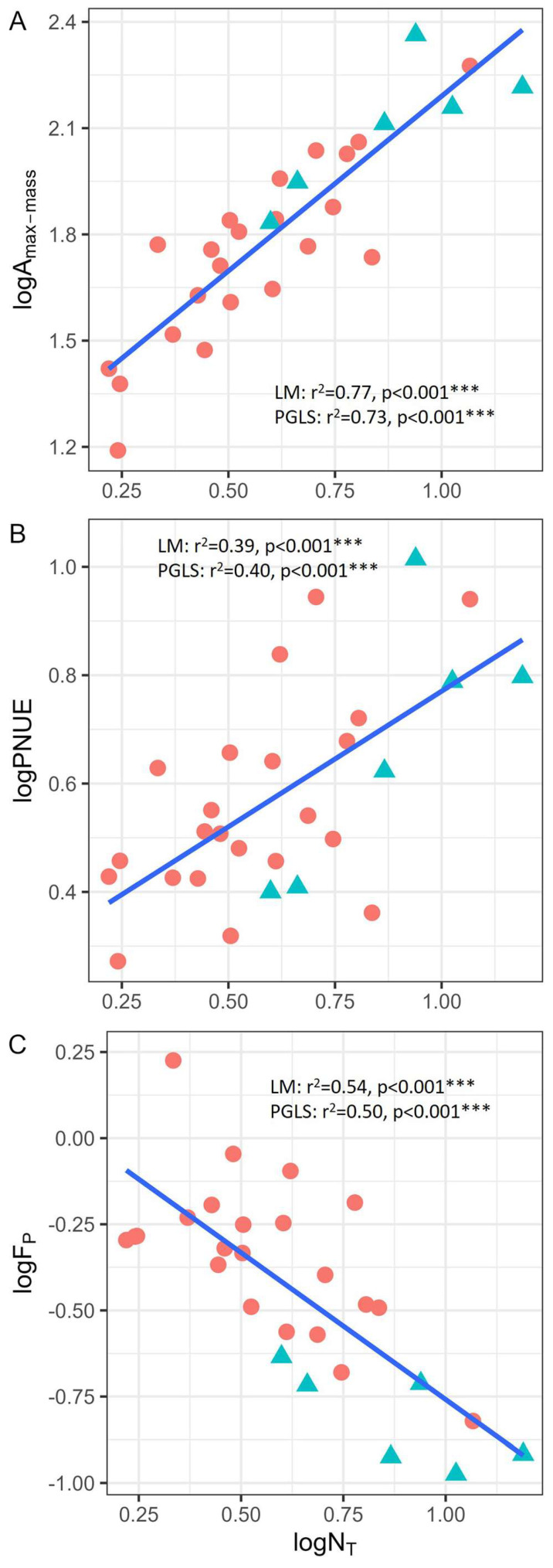
Correlation between leaf nitrogen allocated to photosynthetic apparatus and the maximum photosynthetic rate (**A**), photosynthetic nitrogen-use efficiency (**B**), and leaf punch force (**C**). The adjusted r square and significance of the linear regression model and phylogenetic generalized least squares analysis were separately labelled in each figure. The red dots and green triangles represent the lithophytic and terrestrial orchids, respectively. A_max-mass_: maximum photosynthetic rate; PNUE: photosynthetic nitrogen-use efficiency; *F*_P_: leaf punch force; N_T_: total nitrogen content in photosynthetic apparatus. Statistical significance level is denoted by asterisks (*** *p* < 0.001).

Correlation analysis revealed significant relationships between nitrogen allocation to photosynthesis and traits associated with photosynthesis and biomechanics. Specifically, A_max-mass_ and PNUE were significantly influenced by N_T_ (Figure 3A,B); the larger the N_T_, the larger the A_max-mass_ and PNUE. *F*_P_ was significantly negatively correlated with N_T_ (Figure 3C). These correlations remained significant even after phylogenetic effects were excluded. The results of ANCOVA (Supplementary Table S2) revealed that the interaction between N_T_ and growth form was not significant (*p* > 0.05), indicating that a unified regression equation can be used to describe the correlations between the traits of different growth forms.

### 3.3. Correlations Between Leaf Saturated Water Content and Photosynthesis, Biomechanical Strength, and Leaf Structure

**Figure 4:**
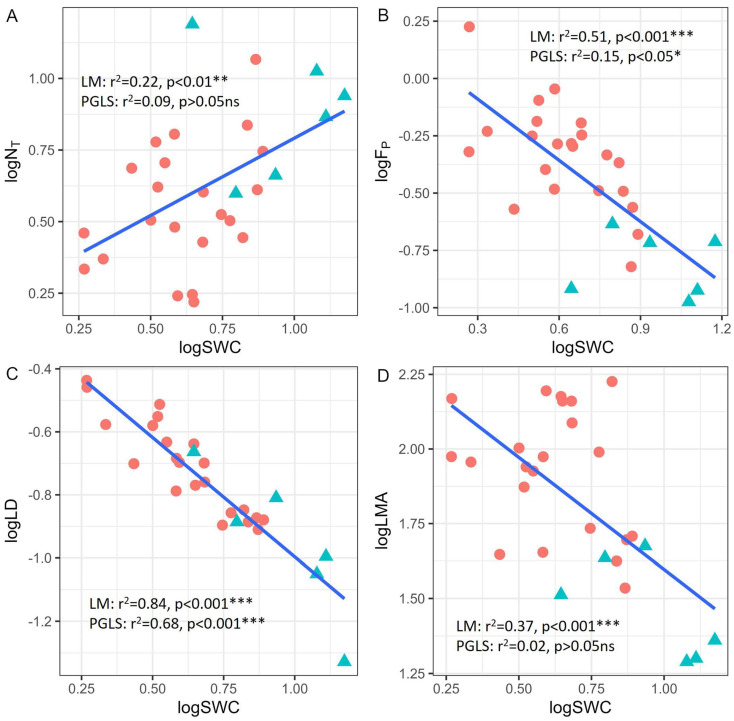
Correlations between leaf saturated water content and total nitrogen content in photosynthetic apparatus (**A**), biomechanical trait (**B**), and leaf structural traits (**C**,**D**). The adjusted r square and significance of the linear regression model and phylogenetic generalized least squares analysis were labelled separately in each figure. The red dots and green triangles represent the lithophytic and terrestrial orchids, respectively. N_T_: total nitrogen content in photosynthetic apparatus; *F*_P_: leaf punch force; LD: leaf density; LMA: leaf mass per area; SWC: leaf saturated water content. Statistical significance levels are denoted by asterisks (* *p* < 0.05, ** *p* < 0.01, *** *p* < 0.001).

Linear regression models revealed significant correlations between leaf saturated water content (SWC) and photosynthesis (N_T_), biomechanical strength (*F*_P_), and leaf structure (LD and LMA). Specifically, SWC was positively correlated with N_T_ (Figure 4A) and negatively correlated with *F*_P_, LD, and LMA (Figure 4B–D). However, after eliminating phylogenetic effects by PGLS, a very weak trade-off relationship was observed between SWC and *F*_P_ (Figure 4B). No significant correlation was found between SWC and N_T_ (Figure 4A) or LMA (Figure 4D), but a strong and significant correlation was observed between SWC and LD (Figure 4C). The ANCOVA results revealed consistent correlations across growth forms (Supplementary Table S2).

## 4. Discussion

### 4.1. Differences in Leaf Traits Between Lithophytic and Terrestrial Orchids

Overall, the results underscore the complex interplay of functional traits in shaping the ecological strategies of orchids in karst environments, suggesting a more conservative ecological strategy for lithophytic orchids. Drought-tolerant plants typically show higher leaf mass per area (LMA) [29], resulting from structural adaptations to water stress. These adaptations include thickened epidermal and palisade tissues [30,31], increased cell wall thickness, and elevated cellulose and lignin content [32], all of which contribute to a higher leaf density and LMA [30,33]. Notably, thicker cell walls enhance resistance to mechanical deformation and significantly increase the bulk elastic modulus (ε) [34], demonstrating a strong positive correlation between the LMA and ε [35]. When external water potential decreases to a fixed value, cells with higher ε exhibit reduced water loss but more pronounced changes in turgor pressure and water potential [36]. This adaptive mechanism helps maintain a substantial water potential gradient, thereby facilitating water uptake in drought-prone environments [37]. Therefore, the significantly higher LMA observed in lithophytic orchids compared to terrestrial species suggests enhanced drought resistance in their leaf tissues. Furthermore, the thicker cuticle characteristic of lithophytes provides additional protection against water loss, contributing to better leaf water balance and drought prevention [38]. Therefore, these structural and anatomical adaptations enhance the drought resistance in lithophytic orchids [39]. As climate change results in increased drought stress, these characteristics of lithophytic orchids may increase their chances of survival in future environments.

The investigated orchids at the study site grow in low-lying soil or on rock surfaces. Under the dense forest canopy, with coverage exceeding 80%, both lithophytic and terrestrial orchids are subjected to consistently low light intensities. Lithophytic orchids had a significantly higher proportion of nitrogen allocated to light-harvesting components (P_L_) and lower Chl_a/b_ than those of terrestrial orchids (Table 1, Supplementary Table S3). A higher P_L_ increases the absorption of light energy [40]. Under low light conditions, the diffuse blue and violet light spectra are predominant, and chlorophyll b is more efficient at absorbing these wavelengths [41]. Lithophytic orchids enhance their ability to capture light energy in shaded environments by increasing light-harvesting components and reducing the chlorophyll a/b ratio, thereby optimizing photosynthesis. The evolution of this mechanism may play a crucial role in helping lithophytic orchids adapt to shaded environments.

The *F*_P_ of lithophytic orchids (0.53 kN m^−1^) is significantly higher than that of terrestrial orchids (0.16 kN m^−1^), surpassing those of woody plants (0.33–0.35 kN m^−1^) and forbs (0.23 kN m^−1^) globally and approach the mean value for monocots (0.56 kN m^−1^) [26]. This value also exceeds the previously recorded value for karst woody plants (0.42 kN m^−1^) [42]. This suggests that lithophytic orchids, particularly those in karst habitats, may have evolved more robust physical defence mechanisms to cope with environmental pressures. Previous studies have demonstrated that shade-tolerant plants generally exhibit extended leaf life span [5,26]. For instance, the leaves of lithophytic *Paphiopedilum* (Orchidaceae) species inhabiting karst environments can persist for over three years [40]. However, their prolonged leaf longevity also increases exposure to various stressors, including insect herbivory, falling branches, animal trampling, and UV light damage. Enhanced *F*_P_ may improve their resilience against such physical threats, thereby better protecting photosynthetic tissues from damage and ensuring sustained, albeit slow, carbon acquisition.

### 4.2. Correlations Among Photosynthesis, Nitrogen Allocation, and Biomechanical Traits

Correlation analysis identified key relationships between nitrogen allocation to photosynthesis and both photosynthetic capacity and leaf biomechanical strength, emphasizing a trade-off between productivity and stress resistance. On average, the A_max-mass_, N_T_, and PNUE of karst orchids were lower compared to those of trees and other herbs [17,43,44,45,46]. Nitrogen is a key element for plant growth and photosynthesis. However, the relationship between photosynthetic capacity and leaf nitrogen content varies significantly across species [47,48], making PNUE a critical factor in driving interspecific differences in photosynthetic capacity [43].

The significant positive correlation between PNUE and N_T_ (Figure 4B) supports previous research findings, which suggest that nitrogen allocation patterns influence PNUE [44,49,50]. The low PNUE in plants could result from the regulation of total cell wall mass, with more nitrogen allocated to cell walls, reducing the nitrogen available for photosynthetic apparatus [51]. A lower N_T_ indicates that less nitrogen is allocated to chloroplasts, which diminishes the availability of key photosynthetic enzymes and inhibits photosynthesis [52]. Therefore, insufficient nitrogen investment in photosynthetic organs may be one of the reasons for the low A_max-mass_ observed in karst orchids.

The theory that PNUE is regulated by nitrogen allocation was further supported by the negative correlation observed in this study between *F*_P_ and N_T_ (Figure 3C). *F*_P_ is a trait closely associated with the cost of leaf construction [26], meaning the trade-off between biomechanics and photosynthesis is influenced by nitrogen allocation. A lower proportion of nitrogen allocated to photosynthesis results in a greater investment in cell walls, which in turn enhances biomechanical strength [4]. These results supported the theory that there is a leaf-level trade-off between productivity and stress resistance associated with nitrogen allocation.

Furthermore, the predictive power of the LMA was stronger in *F*_P_ of karst orchids (PGLS: r^2^ = 0.65, *p* < 0.001) than that of global plant studies (r^2^ = 0.31) [26], and leaf life span was positively correlated with *F*_P_ in woody plants in subtropical forest [5]. Moreover, Onoda et al. suggested that biomechanical strength may be more directly related to leaf life span than the LMA [26]. Biomechanical strength was pivotal in the leaf trait networks of various plants in the karst forest in Southwestern China [42], which may play a central regulatory role affecting the plant phenotype [53]. In addition, the measurement of *F*_P_ is simple and quick, and cheap and simple instruments are used. Therefore, *F*_P_ could predict photosynthetic capacity and leaf life span in future studies on orchid leaf functional traits in karst forests.

### 4.3. Correlation Between SWC and Leaf Traits

In our study, leaf saturated water content (SWC) was strongly correlated with leaf structure (LD) and biomechanical (*F*_P_) traits. These findings contributed to a deeper understanding of the water economy of karst orchids. Leaves with higher water content support enhanced photosynthetic activity and metabolic efficiency [54], traits commonly associated with plants adapted to environments with abundant water supply. However, to maintain higher water content, leaves may compromise investment in structural traits, such as leaf biomechanical strength or density, in order to reduce the mechanical burden. This could be due to a less demanding environment in terms of mechanical stress (e.g., herbivory) or an adaptive avoidance strategy by the plant (e.g., deciduous and dormancy). However, karst habitats are fundamentally characterized by overall water scarcity. The enhanced resistance of lithophytic orchids to the combined stress of drought and herbivory [55] in this region may help explain the dominance of lithophytic orchid communities. Thus, the trade-off between SWC, LD, and *F*_P_ reflects the adaptation of orchids in karst habitats to multiple pressures, including water limitations, physical damage, and photosynthetic efficiency.

Furthermore, no correlation was found between SWC, N_T_, and LMA, which was different from previous findings that reported a positive correlation between leaf water content and the photosynthetic rate [7,48,50]. This discrepancy might be linked to the notably thick upper epidermis (or both epidermal layers) observed in many orchid leaves (Figure S2). These specialized epidermal cells serve as substantial water reservoirs, functionally distinct from the mesophyll tissue responsible for photosynthesis. This means that the water stored in these epidermal cells is not directly utilized for photosynthesis or metabolic processes, thereby decoupling the relationship between leaf water content and the photosynthetic rate. Under drought stress, water loss likely occurs from these non-photosynthetic storage cells—a mechanism that helps preserve the hydration of photosynthetic tissues [36]. This adaptive strategy allows the plant to sustain essential metabolic activities even during periods of limited external water availability [56].

## 5. Conclusions

The research finding demonstrated that compared to terrestrial orchids, lithophytic orchids exhibited a higher LMA and partitioned less nitrogen to the photosynthetic apparatus, resulting in low photosynthetic rates. In accordance with the LES theory, lithophytic orchids employ a slow investment-return strategy. Plants employing such strategies typically exhibit enhanced resistance to environmental stresses [1,57]. The trade-off between photosynthetic capacity and biomechanical strength was consistent across karst orchid growth forms. SWC is a key variable driving the variations in several important traits and thus can be incorporated into the LES of karst orchids. Future work should quantify microenvironmental gradients (e.g., rock vs. soil water retention, light/temperature regimes) to mechanistically explain trait divergence between growth forms.

## Figures and Tables

**Table 1:** Differences in leaf traits between different growth forms of orchid species. A_max-mass_: maximum photosynthetic rate; SD: stomatal density; g_s_: stomatal conductance; PNUE: photosynthetic nitrogen-use efficiency; WUE: water-use efficiency; N_m_: leaf nitrogen content; N_T_: total nitrogen content in photosynthetic apparatus; Chl: leaf chlorophyll content; Chl_a/b_: the ratio of Chl_a_ to Chl_b_; SWC: leaf saturated water content; LMA: leaf mass per area; LDMC: leaf dry mass content; CT: upper cuticle thickness; LT: leaf thickness; LD: leaf density; *F*_P_: leaf punch force. Data are presented as mean ± standard error. Significant differences between different growth forms are presented in bold *p* value.

Variables	Coefficient of Variation	Lithophyte	Terrestrial	*p* Value
A_max-mass_ (nmol g^−1^ s^−1^)	65.26%	64.68 ± 8.37	137.67 ± 23.61	**0.0034**
SD (No. mm^−2^)	50.58%	59.55 ± 6.43	44.84 ± 7.84	0.3954
g_s_ (μmol m^−2^ s^−1^)	49.70%	0.05 ± 0.01	0.05 ± 0.01	0.8665
PNUE (μmol g^−1^ s^−1^)	51.70%	3.97 ± 0.41	5.34 ± 1.20	0.4599
WUE (μmol mol^−1^)	20.79%	96.94 ± 3.72	81.56 ± 9.57	0.1937
N_m_ (mg g^−1^)	37.68%	16.16 ± 1.16	27.40 ± 1.88	**0.0002**
N_T_ (mg g^−1^)	65.53%	4.07 ± 0.49	8.45 ± 1.73	**0.0080**
Chl (mg g^−1^)	36.23%	1.04 ± 0.07	1.61 ± 0.17	**0.0053**
Chl_a/b_	10.50%	2.69 ± 0.06	2.96 ± 0.08	**0.0386**
SWC (g g^−1^)	57.56%	4.53 ± 0.39	9.85 ± 1.67	**0.0034**
LMA (g m^−2^)	57.47%	94.47 ± 9.13	30.90 ± 4.96	**0.0002**
LDMC (g g^−1^)	42.13%	0.20 ± 0.02	0.11 ± 0.02	**0.0034**
CT (μm)	69.95%	2.03 ± 0.27	0.73 ± 0.05	**0.0042**
LT (μm)	54.20%	489.44 ± 53.08	286.15 ± 50.44	**0.0331**
LD (g cm^−3^)	41.11%	0.20 ± 0.02	0.12 ± 0.02	**0.0171**
*F*_P_ (kN m^−1^)	71.26%	0.53 ± 0.07	0.16 ± 0.02	**0.0000**

## Data Availability

The original contributions presented in the study are included in the article/Supplementary Materials; further inquiries can be directed to the corresponding authors.

## Supplementary Materials

The following supporting information can be downloaded at https://www.mdpi.com/article/10.3390/plants14081161/s1. Table S1: List of orchid species used in this study; Table S2: ANCOVA test results for regression slope homogeneity; Table S3: Differences in leaf traits between different growth forms of orchid species. Figure S1: Principal component analysis (PCA) after eliminating those with high collinearity. Figure S2: Leaf transverse sections of 28 orchid species.

## Abbreviations

The following abbreviations are used in this manuscript:

A_max-mass_	Mass-standardized maximum photosynthetic rate
J_max_	Maximum electron transport rate
V_cmax_	Maximum carboxylation rate
Chl	Leaf chlorophyll content
Chl_a/b_	The ratio of Chl_a_ to Chl_b_
CT	Upper cuticle thickness
*F*_P_	Leaf punch force
g_s_	Stomatal conductance
LMA	Leaf mass per area
LDMC	Leaf dry mass content
LD	Leaf density
LT	Leaf thickness
N_m_	Leaf nitrogen content
N_T_	Total nitrogen content in photosynthetic apparatus
PNUE	Photosynthetic nitrogen-use efficiency
SD	Stomatal density
SWC	Leaf saturated water content
WUE	Water-use efficiency
