# Supplementary material for: Different Leaf Strategies Between Lithophytic and Terrestrial Orchids in a Subtropical Karst Forest
Source: Plants (Basel). 2025 Apr 8;14(8):1161. doi: 10.3390/plants14081161 (PMC12030510; doi:10.3390/plants14081161)
Supplement: Supplementary file 1 [file plants-14-01161-s001.zip › plants-3495571-supplementary.pdf]

Supplementary Material

Table S1. List of orchid species used in this study.

No.	Species name	Growth form	Only one leaf for each plant	Have pseudobulb/tuber	$\delta^{13}C$/‰*
1	*Bletilla striata*	Terrestrial	No	Yes	-25.37
2	*Crepidium purpureum*	Terrestrial	No	Yes	-34.30
3	*Habenaria dentata*	Terrestrial	No	Yes	-33.88
4	*Liparis gigantea*	Terrestrial	No	Yes	-28.1
5	*Calanthe argenteostriata*	Terrestrial	No	No	-
6	*Calanthe triplicata*	Terrestrial	No	No	-27.1/-36.1
7	*Bulbophyllum kwangtungense*	Lithophyte	Yes	Yes	-
8	*Bulbophyllum tianguii*	Lithophyte	Yes	Yes	-29.86
9	*Coelogyne flaccida*	Lithophyte	No	Yes	-29.7
10	*Coelogyne fimbriata*	Lithophyte	No	Yes	-28.08
11	*Cymbidium tracyanum*	Lithophyte	No	Yes	~-25.50
12	*Cymbidium floribundum*	Lithophyte	No	Yes	~-29.00
13	*Cymbidium cyperifolium*	Lithophyte	No	Yes	-32.25
14	*Cymbidium lancifolium*	Lithophyte	No	Yes	-32.20
15	*Dendrobium denneanum*	Lithophyte	No	Yes	-25.40
16	*Dendrobium fimbriatum*	Lithophyte	No	Yes	-28.51
17	*Dendrobium chrysanthum*	Lithophyte	No	Yes	-25.40
18	*Eria corneri*	Lithophyte	No	Yes	-27.1
19	*Eria coronaria*	Lithophyte	No	No	-26.70
20	*Eria clausa*	Lithophyte	No	Yes	-29.54
21	*Liparis viridiflora*	Lithophyte	No	Yes	-27.3~-31.4
22	*Liparis esquirolii*	Lithophyte	Yes	Yes	-
23	*Panisea cavaleriei*	Lithophyte	Yes	Yes	-31.43
24	*Paphiopedilum hirsutissimum*	Lithophyte	No	No	-27.51
25	*Paphiopedilum micranthum*	Lithophyte	No	No	-27.53
26	*Paphiopedilum dianthum*	Lithophyte	No	No	-28.26
27	*Pholidota leveilleana*	Lithophyte	Yes	Yes	-26.7~-29.1
28	*Pholidota yunnanensis*	Lithophyte	No	Yes	-30.24

* We searched *Web of Science* and *China National Knowledge Infrastructure* for references to photosynthetic pathways of the included orchids in this study. Of the 28 species, 25 species had $\delta^{13}C$ values that identified them as C_3 plants, and the other three were explicitly defined as C_3 plants in the research papers, although no $\delta^{13}C$ values available. Therefore, all 28 species were classified as C_3 plants.

Table S2. ANCOVA test results for regression slope homogeneity. $A_{max-mass}$: maximum photosynthetic rate; PNUE: photosynthetic N use efficiency; N_T: total nitrogen content in photosynthetic apparatus; SWC: leaf saturated water content; F_P: leaf force to punch; LD: leaf density. P>0.05 indicates that the interaction of independent variables and covariables is not significant.

Dependent variable	Independent variables and covariables	Sum of squares	Degree of freedom	*F* value	*P* value
$A_{max-mass}$	N_T：Growth form	0.0201	1	1.0921	0.3064
PNUE	N_T：Growth form	0.0260	1	1.0728	0.3106
F_P	N_T：Growth form	0.0045	1	0.1498	0.7021
F_P	SWC：Growth form	0.0762	1	2.4190	0.1330
LD	SWC：Growth form	0.0110	1	1.8348	0.1882

Table S3. Differences in leaf traits between different growth forms of orchid species. V_{cmax}: maximum carboxylation rate; J_{max}: maximum electron transport rate; $A_{max-area}$: area standardized maximum photosynthetic rate; SLA: specific leaf area; P_C: the fraction of the total leaf nitrogen allocated to carboxylation; P_B: the fraction of the total leaf nitrogen allocated to bioenergetics; P_L: the fraction of the total leaf nitrogen allocated to light-harvesting components; P_T: P_C+P_B+P_L; N_a: leaf nitrogen content per area. Data are presented as mean ± standard error. Significant differences between different growth forms are presented in bold *P* value.

Variables	Lithophyte	Terrestrial	*P* value
$V_{cmax-area}$ (μmol m^{-2} s^{-1})	28.92±1.17	22.56±2.81	**0.0041**
$J_{max-area}$ (μmol m^{-2} s^{-1})	58.37±3.14	51.86±9.56	**0.0496**
$A_{max-area}$ (μmol m^{-2} s^{-1})	4.98±0.16	3.82±0.18	**0.0014**
SLA (m^2 kg^{-1})	13.46±0.56	36.98±2.00	**0.0000**
P_C	0.18±0.01	0.24±0.02	0.0721
P_B	0.04±0.00	0.06±0.01	**0.0118**
P_L	0.04±0.00	0.03±0.00	**0.0003**
P_T	0.25±0.01	0.33±0.03	0.1190
N_a (g m^{-2})	1.34±0.03	0.87±0.07	**0.0000**

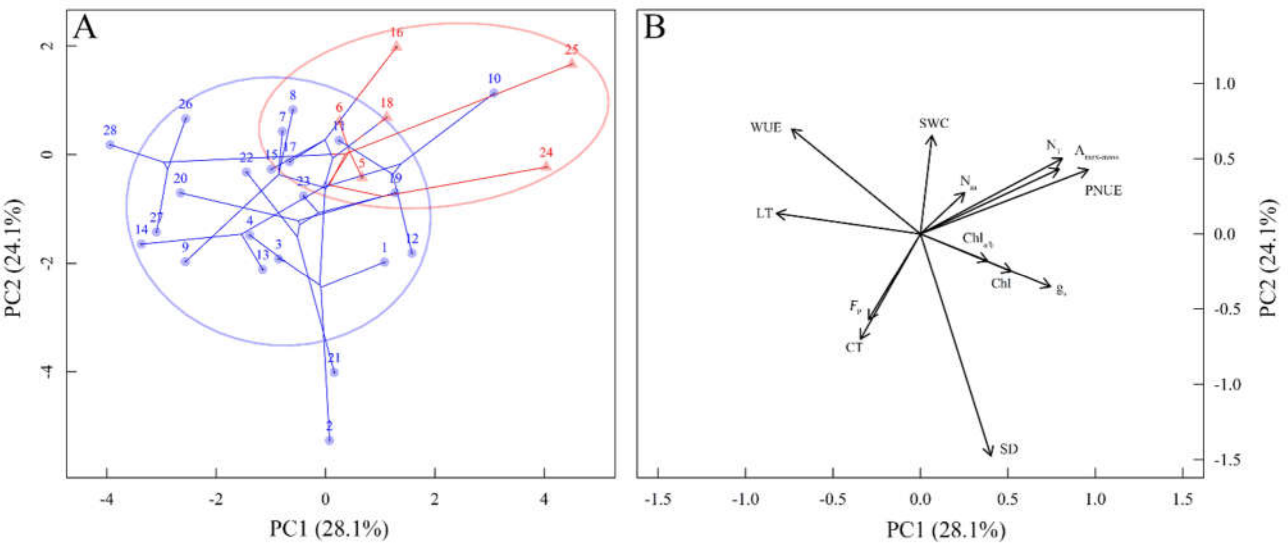

Figure S1: Principal component analysis (PCA) after eliminating those with high collinearity. In this analysis, LMA, LD, LDMC, V_{cmax} and J_{max} were removed. A: Loadings of terrestrial (red triangles) and lithophytic (blue dots) species along PCA axes; B: The correlations between traits and principal components. $A_{max-mass}$: mass-standardised maximum photosynthetic rate; N_m: leaf nitrogen content; N_T: total nitrogen content in photosynthetic apparatus; PNUE: photosynthetic nitrogen use efficiency; Chl: leaf chlorophyll content; $Chl_{a/b}$: the ratio of Chl_a to Chl_b; CT: upper cuticle thickness; LT: leaf thickness; SD: stomatal density; g_s: stomatal conductance; WUE: water use efficiency; SWC: leaf saturated water content; F_P: leaf force to punch.

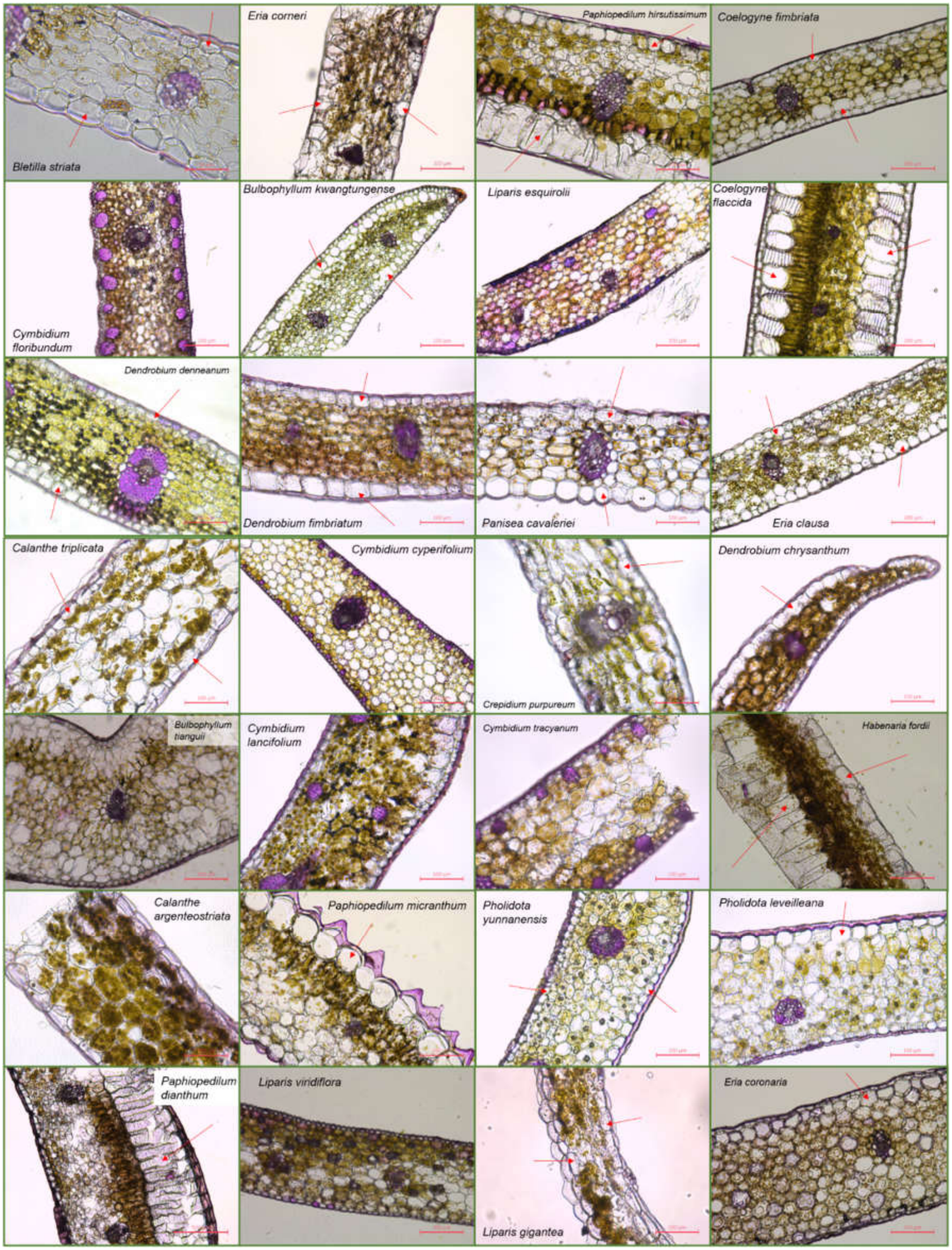

Figure S2. Leaf transverse sections of 28 orchid species. Red arrows highlight the thickened upper and lower epidermal layers.
